# Mr. Frederick Weatherly

**Published:** 1910-08-20

**Authors:** 


Mr. Frederick Weatherly, M.R.C.S., died at Hillside, Portishead, at the age of ninety. Mr. Weatherly was a Bart.'s student, who qualified M.R.C.S., L.S.A., as early as 1841. After forty years of active professional work in the West of England he retired and devoted himself to public work. He was for over thirty years a Poor-Law Guardian, and for many years was chairman of the Bedminster and Long Ashton Boards, and first chairman of the Long Ashton Rural District Council. He was also J.P. for Somerset, and late Surg.-Major 1st Gloucester Volunteer Artillery.

